# NEIGHBORHOOD QUALITY AS IT RELATES TO HEALTH AND WELL-BEING IN OLDER AFRICAN AMERICANS

**DOI:** 10.1093/geroni/igac059.452

**Published:** 2022-12-20

**Authors:** Regina Wright, Alyssa Gamaldo, Anna Lee

## Abstract

With the burgeoning older adult population, there will be an increased demand for neighborhood environments that are conducive to successful aging. For example, the need for affordable and usable housing developments for older adults that provide greater opportunities for social engagement, social services, and convenience to neighborhood resources (e.g., grocery stores, healthcare) will continue to rise. Several initiatives have sought to develop age-friendly neighborhoods, which focused on improving accessibility and affordability of community resources. However, limited effort has focused on the health and cognitive effects of neighborhood-level socioeconomic disadvantage, with respect to neighborhood income, education, employment, and housing quality. This symposium will include presentations from two studies that explored how neighborhood disadvantage (measured by the Area Deprivation Index) relates to health and cognition. The objectives of the proposed symposium are the following: (1) discuss how neighborhood disadvantage relates to subjective and objective measures of physical health; and (2) discuss how neighborhood disadvantage relates to cognitive functioning. Allan and colleagues explored the association between neighborhood disadvantage as it relates to changes in self-reported health and objective measures (i.e., blood pressure) of health among older Black adults. Wright and colleagues explored the association between neighborhood disadvantage and measures of subclinical cardiovascular disease in older Black and White adults. Aiken-Morgan and colleagues examined associations between neighborhood disadvantage and cognitive functioning among Black older adults. Lastly, McCain and colleagues explored associations between neighborhood disadvantage and mobility among Black older adults.

